# A Retrospective Observational Study of COVID-19 Mortality at a Referral Hospital in Fiji

**DOI:** 10.1177/10105395241244461

**Published:** 2024-04-05

**Authors:** Karen Hammad, Yogeshni Chandra, Rigamoto Taito, Manisha Naidu, Ayesha Dean, Yvette Samsioni, Mara Vukivukiseru, Jamie Ranse

**Affiliations:** 1 Menzies Health Institute Queensland, Griffith University, Gold Coast, QLD, Australia; 2 College of Nursing and Health Sciences, Flinders University, Adelaide, SA, Australia; 3 World Health Organization Collaborating Centre for Nursing, Midwifery and Health Development, University of Technology Sydney, Ultimo, NSW, Australia; 4 Fiji Ministry of Health & Medical Services, Toorak, Fiji; 5 Department of Emergency Medicine, Gold Coast Health, Gold Coast, QLD, Australia

## Introduction

The first COVID-19 case in Fiji was a 29-year-old who presented to a hospital on March 18, 2020 with respiratory symptoms. They were immediately isolated until April 22, 2020 following a positive polymerase chain reaction (PCR) test result. For the next 13 months, Fiji reported isolated cases of COVID-19 from returning citizens.^1^ A year later, on April 19, 2021, the first case of community transmission was identified in the Western division, due to a breach in quarantine.^1,2^

Community transmission of COVID-19 in Fiji strained an already fragile health system, including hospitals and clinics. The surge in COVID-19 cases placed immense pressure on the health care system, leading to health care worker infections, overcrowded facilities, and a shortage of resources. These factors combined placed significant pressure on the health system to cope with the normal operational capacity and with the increased workload of patients associated with COVID-19.^2^ Resources such as hospital beds, ventilators, and personal protective equipment (PPE) were reallocated to address the immediate needs of COVID-19 patients. This sometimes resulted in delays or disruptions in the treatment of other medical conditions. The pandemic placed enormous burden on the health workforce. Health workers were at increased risk of contracting COVID-19 leading to quarantines and staff shortages. Overworked health care workers faced physical and mental health challenges, including burnout and stress. The economic impact of the pandemic affected the government’s ability to allocate funds for health care which could have long-term consequences for health care services and infrastructure.

This study aimed to identify opportunities and barriers for the improvement of clinical management and care of people who had COVID-19, who were dead on arrival (DoA) or alive on arrival (AoA) and subsequently died in hospital. The objectives of this research are as follows:

1. Describe the demographic and pre-hospital characteristics of people who had confirmed COVID-19 and who died at or before arrival to the study hospital.
2. Identify and compare known factors associated with higher risk of mortality and severe disease of people who had confirmed COVID-19 and who died at or before arrival to the study hospital.

## Methods

### Design

This retrospective observational study was guided by the Strengthening the Reporting of Observational Studies in Epidemiology Statement.^3^

### Setting

The setting for this study is a divisional hospital in Fiji (“the study hospital”), with 305 beds, making it one of the largest public hospitals in Fiji.

### Population and Sample

The population for this research were all people who presented to the hospital between July 1, 2021 and August 30, 2022. The sample includes those who had COVID-19 on arrival.

### Data Collection

Data were collected from hospital records including patient notes, emergency department referral book, vaccine records, and death certification folder. Data coding occurred to allow some statistical tests assumptions to be met. The category “not documented” was included for completeness; however, it is considered a missing variable. Recoding was undertaken for the variable “risk factors” against the Fiji MHMS in the “COVID-19: Clinical guidelines—adult case management” lists risk factors for severe COVID-19 disease.^4^ Recording of “location prior to arrival” resulted in the category “transferred from other health facility,” referred to people transferred from “subdivisional hospital,” “health centers,” or “aged care.”

### Data Analysis

Data were analyzed using descriptive statistics and non-parametric statistics, as the data were not normally distributed. Chi-square statistics explored differences in proportions, such as “alive on arrival” and “dead on arrival.” Continuous data, such as age, were analyzed using a Mann–Whitney *U* test. Statistical significance was set at *P* ≤ .05.

### Protection of Human Participants

Ethical approval was received the Fiji Human Health Research and Ethics Review Committee (29/2022). Institutional approval to conduct this research was obtained.

## Results

During the study period, 864 people who presented to the study hospital were COVID-19 positive. Of these, there were 257 deaths, including people who were DoA or AoA. We were able to view medical records for 227 of those who died.

### Pre-hospital Characteristics

Pre-hospital characteristics are presented in Table 1. People who were AoA were more likely to have come from a health facility when compared with home (*P* < .001).

**Table 1.** Pre-hospital Characteristics, Prior to Arrival at the Hospital.

	Alive on arrival (n= 118)	Dead on arrival (n= 109)	Total (n= 227)	Statistic
Location prior to arrival, *n (%)*				*P* < .001^a^
Home	44 (37.3)	90 (82.6)	134 (59.0)	
Transferred from other health facility	70 (59.3)	2 (1.8)	72 (31.7)	
Not documented^b^	4 (3.4)	18 (16.5)	22 (9.7)	
Mode of arrival, *n (%)*				N/A
Interfacility transfer	71 (60.2)	2 (1.8)	73 (32.2)	
Police	1 (0.8)	26 (23.9)	27 (11.9)	
Relatives	0	12 (11.0)	12 (5.3)	
Ambulance	1 (0.8)	2 (1.8)	3 (1.3)	
Not documented^b^	45 (38.1)	67 (61.5)	112 (49.3)	

n, number; N/A, not applicable as assumptions are not met for data analysis due to low numbers in some categories.

a Statistical significance.

b Not documented is excluded from analysis.

### Demographic Data

Demographic data relating to those who arrived to hospital are presented in Table 2. Proportionately, more Fijian Indians were AoA when compared with the indigenous Fijian community (*P* < .001). The DoA group were statistically significantly older than those who were AoA (*P* < .001). Of clinical meaningfulness, there are many people from the study sample that had a comorbidity, such as hypertension (n = 104, 45.8%), diabetes (n = 102, 44.9%), (n = 74, 32.5%), or kidney disease (n = 51, 22.4%).

**Table 2.** Demographic Data.

	Alive on arrival (n = 118)	Dead on arrival (n = 109)	Total (n = 227)	Statistic
Sex, *n (%)*				*P* = .45
Male	58 (49.2)	60 (55.0)	118 (52.0)	
Female	60 (50.8)	49 (45.0)	109 (48.0)	
Age in years, *M (IQR)*	60 (54,67)	68 (56,78)	63 (54,72)	*P* = .001^a^
Ethnicity, *n (%)*				*P* < .001^a^
Taukei (Indigenous Fijian)	54 (45.8)	76 (69.7)	130 (57.3)	
Fijian Indian	60 (50.8)	33 (30.3)	93 (41.0)	
Other^b^	4 (3.4)	0	4 (1.8)	
Known comorbidities, *n (%)*^c^				*P* = .075
Hypertension	61 (51.7)	43 (39.4)	104 (45.8)	
Diabetes	64 (54.2)	38 (34.9)	102 (44.9)	
Cardiovascular disease	42 (35.6)	31 (28.4)	73 (32.2)	
Kidney disease	34 (28.8)	17 (15.6)	51 (22.5)	
Other	64 (54.2)	71 (65.1)	135 (59.5)	

IQR, Interquartile range; M, Median; n, number; N/A, not applicable.

a Statistical significance.

b Excluded from analysis as the variable does not meet the statistical assumptions.

c A patient may have had one or more risk factor and/or comorbidity.

### COVID-19 Data and Characteristics

Proportionately, those who had one or two vaccine doses were AoA (*P* = .03). Proportionately, more people were AoA with confirmed COVID-19. Of clinical meaningfulness, the largest proportions of known risk factors had multiple comorbidities (n = 117, 78.0%) and/or were immunocompromised (n = 113, 49.8%) on arrival (Table 3).

**Table 3.** COVID-19 Characteristics.

	Alive on arrival (n = 118)	Dead on arrival (n = 109)	Total (n = 227)	Statistic
Risk factors, *n (%)*				*P* = .56
Age over 50 years	93 (78.8)	87 (79.8)	180 (79.3)	
One or more known comorbidity	97 (82.2)	80 (73.4)	177 (78.0)	
Immunocompromised	67 (56.8)	46 (42.2)	113 (49.8)	
Other	25 (21.2)	15 (13.8)	40 (17.6)	
Smoker	12 (10.2)	7 (6.4)	19 (8.4)	
Not documented^a^	21 (17.8)	13 (11.9)	34 (15.0)	
Immunization status, *n (%)*				*P* = .03^b^
No vaccination	68 (57.6)	78 (71.6)	146 (64.3)	
1 or 2 vaccination doses	50 (42.4)	31 (28.4)	81 (35.7)	
Day from symptom onset and hospital presentation, *M (IQR)*	4 (2.8)^c^	N/A	N/A	
Confirming COVID-19 status, *n (%)*				*P* < .001^b^
Known positive prior to arrival	26 (22.0)	1 (0.9)	27 (11.9)	
Confirmed after arrival	71 (60.2)	104 (95.4)	175 (77.1)	
Missing^a^	21 (17.8)	4 (3.7)	25 (11.0)	

IQR, Interquartile range; M, Median; n, number.

a Excluded from analysis.

b Statistical significance.

c 41 people excluded as they had missing data.

The average time from symptom onset to presentation to hospital was 4 days.

## Discussion

This research aligns with what is known about COVID-19 severity emphasizing that those who are older, have comorbidities, and are unvaccinated remain at risk for severe COVID-19 disease. This research also aligns with what is known about indigenous communities as vulnerable populations. Other key findings from our study identified that most people who died were at home prior to arrival and that for the majority the COVID-19 status was unknown on arrival.

Limitations of this study are that it is difficult to draw specific conclusions as medical records for 11% of patients could not be obtained due to misfiling of paper-based patient medical records.

## Conclusion

The findings from this research are useful in planning for future outbreaks, epidemic and pandemic situations in Fiji, and other Pacific Island Countries. These findings emphasize the need to strengthen risk communication and community engagement during outbreaks to ensure vulnerable populations have access to appropriate health information and health services on time to optimize health outcomes. Clinical pathways including referrals from low-level health facilities to higher-level health facilities need to be strengthened and well communicated to all health care workers and the community to optimize utility and health outcomes. In addition, this research supports the importance of health workers maintaining a high level of suspicion during outbreak situations. Health workers have adequate knowledge to protect themselves as well as access to adequate PPE.
